# Comparison of *BRCA1* Expression between Triple-Negative and Luminal Breast Tumors

**DOI:** 10.22034/ibj.22.3.210

**Published:** 2018-05

**Authors:** Farzaneh Darbeheshti, Pantea Izadi, Amir Nader Emami Razavi, Mir Saeed Yekaninejad, Javad Tavakkoly Bazzaz

**Affiliations:** 1 Department of Medical Genetics, School of Medicine, Tehran University of Medical Sciences, Tehran, Iran; 2 Iran National Tumor Bank, Cancer Institute of Iran, Tehran University of Medical Sciences, Tehran, Iran; 3 Department of Epidemiology and Biostatistics, School of Public Health, Tehran University of Medical Sciences, Tehran, Iran

**Keywords:** Breast cancer, *BRCA1*, Triple-negative breast neoplasm

## Abstract

**Background:**

Previous studies have suggested that *BRCA1* dysregulation has been shown to have a role in triple-negative phenotypic manifestation. However, differences of *BRCA1* expression, as a tumor suppressor gene, have rarely been investigated between luminal and triple-negative breast tumors. Therefore, the present study attempted to compare the *BRCA1* expression in triple-negative with luminal breast tumors and its association with the clinicopathologic characteristics of patients.

**Methods:**

*BRCA1* expression was evaluated by real-time PCR in 26 triple-negative and 27 luminal breast tumors.

**Results:**

The results revealed that there is a high frequency of *BRCA1* underexpression in both triple-negative and luminal breast tumors. The *BRCA1* underexpression was related to young age at diagnosis, lymph node involvement, and grade III tumors.

**Conclusion:**

The observations suggest that decreased *BRCA1* expression, regardless of tumor subtype, has a general role in breast malignancy and associated with poor prognostic features in breast tumors.

## INTRODUCTION

Breast cancer is the most common cancer among women worldwide[1]. In Iran, breast malignancy is the fifth most common cause of death with a fast-rising trend[2]. Breast tumors show different molecular features and can be divided into at least four main molecular subtypes: luminal A and B as well as triple-negative and HER2-overexpressing tumors[3]. Luminal tumors are estrogen receptor positive (ER+), progesterone receptor-positive (PR+), and positive or negative for human epidermal growth factor receptor 2 (HER2+ or HER2-). These tumors have a good prognosis and respond to targeted therapies such as tamoxifen. On the other hand, triple-negative tumors (ER-/PR-/HER-) show aggressive behavior and a worse prognosis in comparison with other subtypes[4].

*BRCA1* is one of the genes that involves in breast cancer. The protein product of the *BRCA1* gene is a 220-kD nuclear phosphoprotein with 1863 amino acids and different important cellular functions. *BRCA1* protein helps to repair DNA double-strand breaks and plays a critical role in maintaining the genomic stability, cell cycle regulation, and apoptosis[5,6]. Accordingly, *BRCA1* deficiency can activate the tumorogenesis process. The association between germline mutations of *BRCA1* and hereditary form of breast cancers is well known[7,8].

Previous investigations have shown that the majority of *BRCA1*-mutated breast tumors (over 80%) are categorized as triple-negative subtype[9]. Furthermore, several studies have demonstrated that the reduced levels of *BRCA1* expression, due to promoter hypermethylation or somatic mutation, may take a part in sporadic breast cancers[10-12]. Interestingly, the sporadic *BRCA1*-deficient breast tumors often show similar histological characteristics with the *BRCA1*-related hereditary breast cancers[13-15]. Therefore, it seems that the dysfunctional *BRCA1* pathway has a function in the manifestation of the triple-negative phenotype in breast tumors[16-18]. However, the comparison of *BRCA1* mRNA expression between different subtypes of breast tumors is rarely available. It is unclear that *BRCA1* down-regulation is a prominent feature of triple-negative breast tumors, or it must be noticed as a more general molecular alteration in breast cancer regardless of tumor subtype. Therefore, the aim of the present investigation was to compare *BRCA1* expression in the setting of triple-negative and luminal tumors and to study the association of *BRCA1* expression with clinicopathological features in Iranian breast cancer patients.

## MATERIALS AND METHODS

### Patients and tissue collection

A total of 53 surgically resected breast tumors were obtained from the Iran National Tumor Bank (INTB) of the Cancer Institute at Imam Khomeini Hospital Complex, Tehran, Iran. As calibrator samples, four normal breast tissues were acquired from women who were undergoing mammoplasty. Tissues were placed in liquid nitrogen immediately after resection and stored at -80 °C for later use. None of the patients were under chemotherapy or radiotherapy before surgery. Clinico-pathological features of the patients (age, tumor size, ER/PR/HER2 status based on immunohistochemistry results, axillary lymph node involvement, and grade) were collected from their medical records in INTB. Informed consents were obtained from all participants, and the study was approved by the local ethical committee at Tehran University of Medical Sciences, Iran.

### RNA extraction and cDNA synthesis

Total RNA was isolated from tissues using Hybrid-R™ kit from GeneAll Biotechnology Company (Korea) according to the manufacturer’s instructions. The purity and quantity of extracted RNA were checked by NanoDrop 2000 Spectrophotometer (Thermo Scientific, USA). Hyperscript™ kit (GeneAll Biotechnology Co., Korea) was applied to synthesize first-strand cDNA.

### Gene expression study

Real-time quantitative RT-PCR of the *BRCA1* gene was performed using RealQ Plus 2× Master Mix Green (Ampliqon, Denmark) following the manufacturer’s instructions. The primers sequence for the *BRCA1* mRNA expression assay were: forward 5´-CCCTCAA GGAACCAGGGATG-3´ and reverse 5´-GCTGCA CGCTTCTCAGTGGT-3´. *BRCA1* expression levels were normalized against *PUM1* (Pumilio RNA-binding family member 1), as a housekeeping gene. The primers for *PUM1* mRNA expression assay were: forward 5´-AGTGGGGGACTAGGCGTTAG-3´ and reverse 5´-GTTTTCATCACTGTCTGCATCC-3´.

The real-time PCR reaction mix consisted of 10 μL SYBR Green master mix, 0.5 μL of each forward and reverse primers (primer concentration: 5 pmol), 1 μL target cDNA, and 8 μL sterile water in a total volume of 20 μL. The PCR conditions were as follows: initial denaturation at 95 °C for 15 minutes, followed by 40 cycles of 95 °C for 15 seconds, and 59 °C for 60 seconds. Four normal breast tissues were used as the calibrator for obtaining relative expression between breast tumors and normal breast tissues (2^-ΔΔCT^ method)[19]. As the range of *BRCA1* expression values in four normal breast tissues was 0.51 to 2.38, the values of ≥2.5 and ≤0.4 were considered overexpression and underexpression status, respectively, in breast tumors.

### Statistical analysis

Statistical calculations were performed using SPSS 21 statistical software. Data were presented with mean and 95% CI for numerical data or percentage for qualitative data. Student’s *t*-test was performed to analyzedifference in *BRCA1* expression between luminal and triple-negative tumors. The relationship between *BRCA1* relative expression and clinicopathologic factors were assessed by *t*-test or ANOVA and alternative non-parametric tests. Differences were considered significant when *p* < 0.05 was obtained.

## RESULTS

### Clinicopathological characteristics

In a total of 53 samples, 26 and 27 breast cancer patients were classified as triple-negative and luminal (types A or B) subtypes, respectively. The tumors were divided into different groups according to age (<50 years: 31 tumors; ≥ 50 years: 22 tumors), size (≤2 cm: 3 tumors; 2-5 cm: 44 tumors; >5 cm: 6 tumors), grade (I, II: 26 tumors; III: 27 tumors), and nodal status (positive: 19 tumors; negative: 34 tumors).

### Expression status of BRCA1 mRNA in breast tumors

As the expression of *BRCA1* mRNA values for all four normal breast samples were between 0.51-2.38, values of ≥2.5 were considered as the overexpression t status and those of ≤0.4 as underexpression status in breast tumors. The frequency of different statuses of *BRCA1* mRNA expression in luminal and triple-negative tumors is indicated in Table 1.

**Table 1:** *BRCA1* mRNA expression in breast tumors

*BRCA1* mRNA expression status	Total (%) (n = 53)	Triple-negative tumors (%) (n = 26)	Luminal tumors (%) (n = 27)
Underexpression	67.9	73.1	63.0
Normal expression	28.3	26.9	29.6
Overexpression	3.8	0.0	7.4

### Comparison of BRCA1 expression between triple-negative and luminal subtypes

**Fig. 1:**
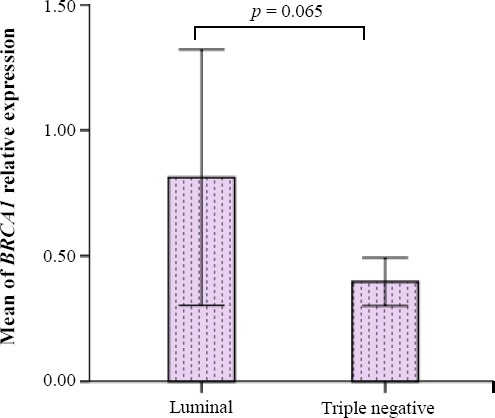
Differences in theexpression of *BRCA1* in luminal and triple-negative tumors. Data were normalized to Pumilio RNA-binding family member 1expression, as a housekeeping gene (error bars: 95% CI).

Independent samples *t*-test showed that the means of *BRCA1* mRNA relative expression were not significantly different (*p* = 0.065) between luminal and triple-negative subtypes (Fig. 1). Also, in triple-negative tumors, *BRCA1* relative expression (mean mean ± SD: 0.26 ± 0.32 showed a decreased level in comparison with luminal tumors (mean ± SD: 1.3 ± 1.5).

### BRCA1 mRNA expression and its clinico-pathological significance

Independent samples *t*-test in 53 breast tumors indicated that decreased expression of *BRCA1* significantly related to young age at diagnosis (< 50 years, *p* = 0.028), lymph node involvement (*p* = 0.04), and grade III (*p* = 0.04) in breast tumor samples, but it did not significantly associate with tumor size and ER/PR/HER2 status of the studied population.

## DISCUSSION

The dysfunctional *BRCA1* pathway is involved in the pathogenesis of both hereditary and sporadic breast cancers. The *BRCA1*-related hereditary breast cancers show a trend toward triple-negative phenotype[9]. Furthermore, decreased *BRCA1* expression, due to promoter hypermethylation or somatic mutations, have been reported in sporadic breast cancers, regardless of breast tumor subtypes[11,12]. As *BRCA1*-deficient sporadic triple-negative tumors show the same histological characteristics as *BRCA1*-related hereditary breast cancers[13,15], it has been suggested that *BRCA1*-deficiency has a role in inducing the triple-negative phenotype. However, the difference in *BRCA1* expression levels, based on the tumor subtypes, has rarely been reported. Accordingly, in the current study, the *BRCA1* mRNA expression was compared in the setting of triple-negative and luminal tumors, and clinicopathological significance of *BRCA1* expression was evaluated in Iranian breast cancer patients.

The results of this study demonstrated that the *BRCA1* underexpression is slightly different between triple-negative and luminal tumors (73.1% and 63%, respectively). This observation suggests that decreased *BRCA1* expression is frequent not only in triple-negative but also in luminal breast cancer tumors. Consequently, *BRCA1* deficiency has possibly a key role in breast malignancy process, apart from tumor subtypes. Interestingly, in our studied patients, *BRCA1* overexpression was observed in two luminal tumors, which belonged to patients with older age at diagnosis (60 and 81 years) and low-grade breast tumors.

Our study revealed that *BRCA1* expression is not significantly different between triple-negative and luminal tumors, though triple-negative tumors overally show a trend to more decrease in *BRCA1* expression as compared to luminal tumors (*p* = 0.065). An investigation on Japanese patients indicated that *BRCA1* mRNA expression is significantly decreased in triple-negative rather than luminal tumors[20].

In this study, decreased expression of *BRCA1* significantly associated with young age at diagnosis, high grade, and lymph node-positive tumors. It seems that the decrease in *BRCA1* expression, whether due to germline mutations in hereditary breast cancers or hypermethylation in sporadic breast cancers, could increase the risk of breast cancer in women at younger ages[21,22]. In several previous studies, it has been demonstrated that the lower level of *BRCA1* expression, as a tumor suppressor gene, was associated with poor prognostic features[21-25]. On the other hand, some studies did not find any association between *BRCA1* mRNA expression and clinicopathological characteristics[11,26-28], suggesting a more complex molecular association. For instance, Egawa *et al*.[26] suggested that decreased *BRCA1* expression alone might not be enough for the development of poor prognostic features, and additional genetic alterations such as *p53* abnormality might be necessary.

In conclusion, in the present study, the decreased levels of *BRCA1* mRNA expression in the majority of triple-negative and luminal tumors compared to normal breast tissues indicates the involvement of *BRCA1* even in luminal subtype, though down-regulation of *BRCA1* expression was more remarkable in triple-negative tumors.
